# Cationic bactericidal peptide 1018 does not specifically target the stringent response alarmone (p)ppGpp

**DOI:** 10.1038/srep36549

**Published:** 2016-11-07

**Authors:** Liis Andresen, Tanel Tenson, Vasili Hauryliuk

**Affiliations:** 1 Department of Molecular Biology, Umeå University, Building 6K, 6L University Hospital Area, SE-901 87 Umeå, Sweden; 2 Laboratory for Molecular Infection Medicine Sweden (MIMS), Umeå University, Building 6K and 6L, University Hospital Area, SE-901 87 Umeå, Sweden; 3 University of Tartu, Institute of Technology, Nooruse 1, 50411 Tartu, Estonia

## Abstract

The bacterial stringent response is a key regulator of bacterial virulence, biofilm formation and antibiotic tolerance, and is a promising target for the development of new antibacterial compounds. The intracellular nucleotide (p)ppGpp acts as a messenger orchestrating the stringent response. A synthetic peptide 1018 was recently proposed to specifically disrupt biofilms by inhibiting the stringent response via direct interaction with (p)ppGpp (de la Fuente-Núñez *et al*. (2014) PLoS Pathogens). We have interrogated the specificity of the proposed molecular mechanism. When inhibition of *Pseudomonas aeruginosa* planktonic and biofilm growth is tested simultaneously in the same assay, peptides 1018 and the control peptide 8101 generated by an inversion of the amino acid sequence of 1018 are equally potent, and, importantly, do not display a preferential activity against biofilm. 1018 inhibits planktonic growth of *Escherichia coli* equally efficiently either when the alleged target, (p)ppGpp, is essential (MOPS media lacking amino acid L-valine), or dispensable for growth (MOPS media supplemented with L-valine). Genetic disruption of the genes *relA* and *spoT* responsible for (p)ppGpp synthesis moderately sensitizes – rather than protects – *E. coli* to 1018. We suggest that the antimicrobial activity of 1018 does not rely on specific recognition of the stringent response messenger (p)ppGpp.

IDR-1018 (or just 1018) is a small cationic synthetic peptide (VRLIVAVRIWRR-NH_2_) that has been developed based on bactenecin, a peptide antibiotic isolated from bovine neutrophil granules^1^,^2^,^3^. This peptide has numerous biological activities that target both eukaryotic and bacterial cells (reviewed by Mansour and colleagues^4^). By targeting the eukaryotic host it acts as a modulator of the immune system, affecting macrophage polarization^5^,^6^ and reducing levels of lipopolysaccharide-induced cytokine production^3^,^7^,^8^. By targeting both Gram-negative (e.g. *Pseudomonas aeruginosa* and *Escherichia coli*) and Gram-positive (e.g. *Staphylococcus aureus*) bacteria 1018 acts as a potent antibacterial: it kills bacteria, disperses biofilms and inhibits bacterial swarming^9^,^10^. Potential practical applications of 1018 range from neuroprotection^8^ and wound healing^11^ to potentiation of antimalarial^12^, antiviral^13^ and antibacterial compounds^14^.

It has been proposed that the dispersal of biofilms by 1018 is mediated by a complex formation between the peptide and the intracellular alarmone nucleotides guanosine tetraphosphate (ppGpp) and pentaphosphate (pppGpp)^9^. The complex formation, in turn, triggers degradation of the nucleotides via a presently unidentified mechanism. The two alarmones, collectively known as (p)ppGpp, are key physiological regulators of the stringent response^15^, which, in turn, is a key regulator of bacterial virulence and antibiotic tolerance^16^,^17^,^18^. In this study we analyse the specificity of peptide 1018 in its effect against *P. aeruginosa* PAO1 planktonic and biofilm growth, as well as its interaction with ppGpp.

## Results

### Peptide 1018 is equally efficient against biofilm and planktonic populations, as judged by crystal violet staining assay

In the original report by de la Fuente-Núñez and colleagues, 1018 was shown to be relatively inefficient against planktonic *P. aeruginosa*, with a minimal inhibitory concentration (MIC) of 41 μM (64 μg/ml)^9^ or 19 μM (29.7 μg/ml)^3^,^19^, depending on the exact method used for the determination of the MIC. It was, however, considerably more potent against biofilms, completely inhibiting their establishment at 6.4 μM (10 μg/ml) (MBIC_100_, minimal biofilm inhibitory concentration 100%), as well as killing an established biofilm at the same concentration. Moreover, 1018 dispersed biofilms at a concentration as low as 0.5 μM (0.8 μg/ml). Since, in general, biofilms are much more resistant to antibacterial compounds than planktonic bacteria^20^, the authors have concluded that an unusual molecular mechanism of action of 1018 is responsible for the peptide specifically targeting biofilms.

An alternative explanation is that 1018 was performing better in the biofilm assay simply due to the way the assay was set up. Effects on biofilms were studied on glass in flow cell chambers under conditions of constant medium flow^10^. This would allow for the sorption and accumulation of the peptides on the cell surface over time, and surface-specific sorption is a well-known property of cationic peptides^21^. The MIC was determined using a broth microdilution method, which does not involve a continuous flow of liquid and is performed with 96-well plates or Eppendorf tubes made of laboratory plastic^22^. Finally, the antibacterial efficiency is strongly affected by bacterial density^23^, which can potentially differ in two separate assays.

**Figure 1:**
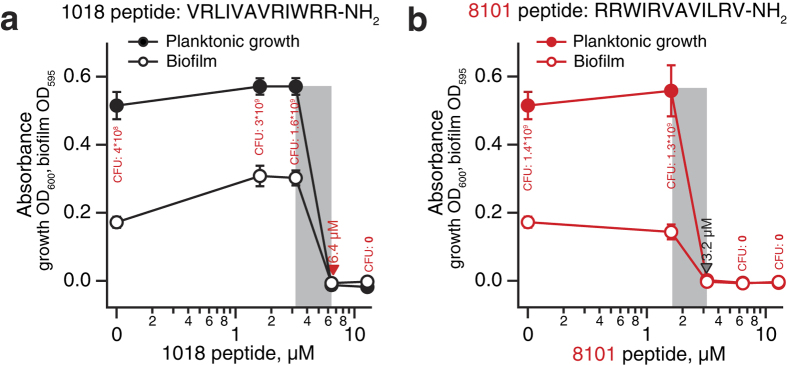
Peptide 1018 (**a**) and its inverted version 8101 (**b**) inhibit *P. aeruginosa* PAO1 growth and biofilm formation equally efficiently. Planktonic growth at 600 nm (filled cycles) was measured from the cultures taken from the same plates that were used later for biofilm measurements. Biofilm formation (open circles) was determined using a crystal violet staining assay^24^ where the intensity of the violet colour corresponds to the biofilm thickness measured spectrophotometrically at 595 nm (open cycles). Highlighted area indicates peptide concentration range where it becomes lethal. Cell count measurements are expressed in colony forming units, CFU, per ml. The results are shown as mean values ± SD of two biological replicates, each estimated from three technical replicates.

In order to circumvent the above-mentioned issues we opted for a robust and widely adopted crystal violet (CV) staining assay that can measure both planktonic growth and biofilm formation in the same well of a 96-well plate^24^,^25^. First, the planktonic population is measured by removing half of the culture form the well, measuring the absorbance at 600 nm. Second, the remaining liquid is aspirated, the biofilm is stained with CV dye and the absorbance at 595 nm is quantified. Similarly to de la Fuente-Núñez and colleagues^9^, we used *P. aeruginosa* strain PAO1. We do not observe a specific anti-biofilm effect: both planktonic and biofilm growth are completely inhibited by 1018 at 6.4 μM (Fig. 1a).

### Inversion of the amino acid sequence of 1018 improves its antimicrobial properties

Peptide 1018 was suggested to exert its anti-biofilm effect by means of entering the cell, directly binding to the intracellular messenger ppGpp − a highly complex and chiral molecule − and targeting it for degradation via a presently unidentified mechanism^9^. In the follow-up report, de la Fuente-Núñez and colleagues tested several stereoisomeric variants of 1018^26^. Several of the compounds were equally as or more efficient than the parental compound, which is surprising given the importance of stereospecificity in biomolecular interactions in general and drug design in particular^27^,^28^. To further test the structure–activity relationship of 1018, we tested its inverted version (RRWIRVAVILRV-NH_2_), which was designated “8101”. The inverted peptide 8101 inhibits planktonic and biofilm formation even more efficiently then the parental compound, completely preventing bacterial growth at 3.2 μM (Fig. 1b). Similar to 1018, 8101 shows no preferential anti-biofilm activity in the CV assay. Cell count measurements expressed in colony forming units (CFU) per ml corroborate the OD_600_ estimates of cell density. A possible explanation for the similar functional activity of the two compounds is that inverted peptide 8101 retained the 3D structure of the parental 1018, just like in the context of larger proteins where short helical fragments, upon inversion, tend to retain the original structure^29^,^30^.

### Antimicrobial activity of 1018 against *E. coli* does not depend on conditional essentiality of its alleged molecular target (p)ppGpp

**Figure 2:**
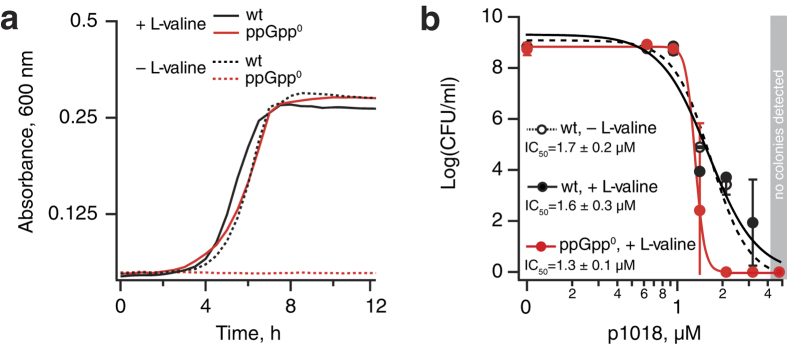
Antimicrobial activity of 1018 against *E. coli* is insensitive to conditional essentiality of the peptide’s alleged molecular target, (p)ppGpp. (**a**) Growth curves of wild type and ppGpp^0^ (Δ*relA*Δ*spoT*) BW25113 *E. coli* in conditions in which (p)ppGpp is essential (MOPS-based minimal medium lacking L-valine) or dispensable with no significant growth defect (MOPS-based minimal medium containing the full set of 20 amino acids). (**b**) Effects of increasing concentrations of 1018 on bacterial survival of wild type BW25113 *E. coli* in MOPS minimal medium with or without addition of L-valine and ppGpp^0^ strain in MOPS supplemented with L-valine. The results are shown as geometric mean values ± SD of two biological replicates, each estimated from two technical replicates. Inhibition efficiency (IC_50_) was calculated using 4-parameter logistic model (Hill equation) as per Sebaugh^48^.

Severity of bacterial phenotype upon genetic disruption of the RSH genes mediating the (p)ppGpp production is dramatically affected by the growth medium^31^,^32^. The growth curves of wild type and ppGpp^0^ Δ*relA*Δ*spoT* in MOPS minimal medium containing L-valine are virtually indistinguishable; conversely, in MOPS medium lacking L-valine ppGpp^0^ stain is unable to grow due to valine auxotrophy^32^,^33^ (Fig. 2a).

We exploited this conditionality of (p)ppGpp’s essentiality for bacterial growth to directly test the proposed mechanism of action of 1018: if, as per de la Fuente-Núñez and colleagues^9^, 1018 acts via direct degradation of intracellular (p)ppGpp, its efficacy should be dramatically different in conditions where the alleged molecular target is essential (MOPS medium without L-valine) or disposable without significant effects on growth rate (MOPS medium supplemented with L-valine). By the same argument, ppGpp^0^ Δ*relA*Δ*spoT* strain lacking (p)ppGpp grown in MOPS medium supplemented with L-valine should be protected from 1018 due to the complete absence of the alleged molecular target of the peptide. However, the killing efficiency of 1018 is similar in all three cases: while at 1 μM the peptide does not affect the cell count, already at 5 μM we detect no viable bacteria (Fig. 2b). The ppGpp^0^ strain is moderately sensitized to 1018 as compare to wild type (IC_50_ 1.3 ± 0.1 μM vs IC_50_ 1.7 ± 0.2 μM) despite the complete lack on the alleged target, (p)ppGpp. Analogous auxotrophy-based tests performed using Gram-positive *Bacillus subtilis* also detect no difference in the killing efficiency of 1018 in conditions when (p)ppGpp is essential or not^34^. Taken together, these results reinforce the idea that the antimicrobial activity of 1018 does not rely on a specific recognition of stringent response messenger (p)ppGpp.

### Peptides 1018 and 8101 co-precipitate with ppGpp in a buffer-specific manner

The direct and specific interaction between 1018 and ppGpp was suggested on the bases of co-precipitation of the two compounds^9^. The original report used 1018 at 250 μM, which is almost two orders of magnitude higher than the concentration used in microbiological assays. We reproduced the experiments with more physiologically relevant concentrations of the peptide by taking advantage of more sensitive detection of the precipitation of tritium-labeled ppGpp (^3^H-ppGpp) by using liquid scintillation counting.

**Figure 3:**
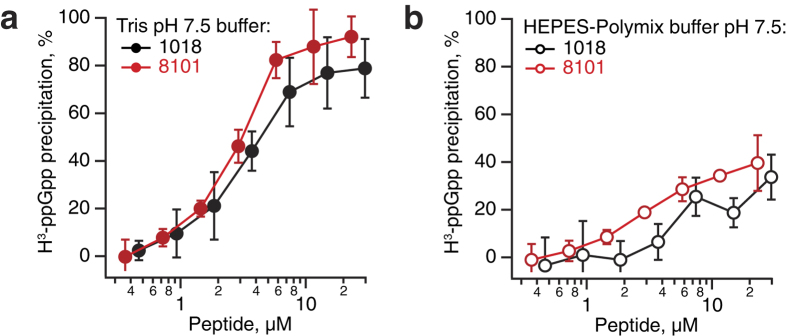
Peptides 1018 and 8101 co-precipitate with ppGpp in a buffer-specific manner. Tritium-labelled 4.5 μM H^3^-ppGpp was mixed with increasing concentrations of peptide 1018 or 8101 in 50 mM Tris-HCl pH 7.5 (**a**) or in HEPES-Polymix (**b**) buffer. After incubation for 10 minutes at room temperature the insoluble material was removed by centrifugation and the radioactivity was quantified by liquid scintillation counting. The results are shown as mean values ± SD of three technical replicates.

First, we titrated both 1018 and 8101 in the presence of 4.5 μM ^3^H-ppGpp in the buffer that was used in the original report (50 mM Tris pH 7.5 buffer^9^) (Fig. 3a). The buffer lacks the essential components that ensure the solubility of biological samples, i.e. inorganic salts that provide the ionic strength, as well as divalent metal ions (e.g. Mg^2+^) and polyamines (e.g. spermidine) that serve as counter-ions and ligands. Almost 40% of ^3^H-ppGpp precipitates at a 1:1 nucleotide-peptide ratio for both peptides and a further increase in peptide concentrations resulted in the precipitation of most of the nucleotides from the solution. However, when we repeat the experiment in the more physiological realistic HEPES-Polymix buffer used to study bacterial protein synthesis^35^,^36^, the peptides are dramatically less efficient in co-precipitating with ppGpp, although there is, again, no difference between the two peptides (Fig. 3b).

## Discussion

Peptide 1018 and its inverted version 8101 are equally efficient in eliminating both planktonic and biofilm *P. aeruginosa* as judged by crystal violet (CV) staining assays (Fig. 1). This suggests the possibility that the preferential effect against biofilms observed by de la Fuente-Núñez and colleagues^9^ could be the result of a flow-chamber detection method favouring inhibition of biofilms by hydrophobic cationic peptides. Current High Throughput Screening protocols for the discovery of anti-biofilm compounds rely on either CV staining in a 96-well format^37^ or flow chambers^10^ for detection. It is possible that the latter approach could overestimate the real potency of these compounds.

While several biochemical and microbiological interactions between 1018 and ppGpp are well documented, one should be cautious in putting forward a specific molecular mechanism of action. In the test tube both 1018 and its inverted version 8101 precipitate ppGpp equally well (Fig. 2). Therefore, specificity towards ppGpp is questionable, and it is likely that general physicochemical properties such as charge and hydrophobicity are at play. Moreover, peptide 1018 co-precipitates with other nucleotides with an efficiency correlating with the number of phosphate groups present in the nucleotide molecule, but not with the nature of the base, i.e. it co-precipitates GTP equally efficiently as ATP, and GDP equally efficiently as ADP^9^.

Induction of the stringent response renders *P. aeruginosa* more tolerant to 1018^9^, which was interpreted as an indication of a specific mechanistic connection between the two. However, the stringent response renders *P. aeruginosa* more resilient in general: it becomes tolerant not only to various antibiotics^38^,^39^,^40^, but also to environmental challenges such as hydrogen peroxide^38^ and UVA^41^ exposure. However, this does not suggest a mechanistic interaction between ppGpp and the stress factors: while accumulation of (p)ppGpp protects from β-lactam ampicillin, (p)ppGpp is not the molecular target of ampicillin – cell wall synthesis is. Degradation of cellular ppGpp upon exposure to 1018 observed by de la Fuente-Núñez and collegues^9^ is, again, not necessarily decisive evidence of the two compounds interacting inside the cell since the alarmone nucleotide is highly labile and rapidly degraded by SpoT hydrolase upon antibacterial treatment with, for example, the antibiotic chloramphenicol^42^.

## Conclusions

While peptide 1018 is a potent antimicrobial, it does not specifically disrupt biofilms via a direct and specific interaction with the intracellular messenger nucleotide (p)ppGpp.

## Methods

### Peptides

Peptides 1018 (VRLIVAVRIWRR-NH_2_) and its inverted version 8101 (RRWIRVAVILRV-NH_2_) were ordered in lyophilized format from Storkbio Ltd and Nordic BioSite AB, respectively (both >95% pure). Peptides were dissolved in water and stored at −80 °C in glass vials. Prior usage peptide concentrations were re-measured according to tryptophan fluorescence (extinction coefficient 5690 M^−1^ cm^−1^) to make sure non-inverted and inverted version of the peptide was used in same concentrations.

### Bacterial strains and growth media

*E. coli* BW25113 wild type (*lacI*^q^ *rrnB*_T14_ Δ*lacZ*_WJ16_ *hsdR514* Δ*araBAD*_AH33_ Δ*rhaBAD*_LD78_) and isogenic ppGpp^0^ Δ*spoT*Δ*relA* strain were described in Jõers and Tenson^43^. *P. aeruginosa* wild type PAO1 was described earlier by Holloway^44^. MOPS supplemented 0.4% glucose was prepared as per Neidhardt *et al*.^45^, except for the omission of thiamine and supplementation with 20 common amino acids (600 μg/ml L-serine, 100 μg/ml L-aspartate, 100 μg/ml L-glutamic acid, 40 μg/ml for others).

### Crystal violet staining assay

Biofilm formation was determined using a crystal violet staining assay as per O’Toole^24^. Wells of a 96-well plate were inoculated with 10^5^ cells in BM2-glucose minimal medium^46^ and increasing concentrations of peptides. After 24 hours at 37 °C, 100 μl of cell suspension was removed from the well and was used for the evaluation of planktonic growth at 600 nm. Adherent biofilms were washed with distilled water and stained with 0.1% crystal violet solution for 20 minutes. Unbound stain was removed with distilled water and biofilm-attached crystal violet was dissolved with ethanol:acetone solution (4:1; vol:vol) for 10 minutes. The intensity of the violet colour corresponding to the biofilm thickness was measured spectrophotometrically at 595 nm.

### Determination of peptide 1018 efficiency against *E. coli*

For 1018 efficiency measurements against *E. coli* BW25113 strains fresh colonies were suspended in MOPS medium lacking L-valine, OD_600_ of the suspension was adjusted to 1.0, and diluted 1000 times for inoculating MOPS medium, with or without L-valine. 10^4^ CFUs per well were seeded in 96-well plates (Sarstedt) in presence of increasing concentrations of 1018 and grown aerobically at 37 °C for 8 hours followed by the CFU measurements.

### Colony forming unit (CFU) measurements

10x serial dilution series of the bacterial cultures were prepared in PBS buffer and 5 μl of each suspension was spotted on dry LB plates. After an overnight incubation at 37 °C colonies were counted and CFU/ml calculated. Detection limit for CFU measurements was ≈500 CFU/ml.

### ppGpp precipitation assay

Tritium-labelled 4.5 μM ppGpp was mixed with increasing concentrations of peptide 1018 or 8101 in 50 mM Tris-HCl pH 7.5 or in HEPES-Polymix^35^,^36^ buffer. After incubation for 10 minutes at room temperature the insoluble material was collected by centrifugation for 5 minutes at 16,000 g and the radioactivity was quantified by liquid scintillation counting. H^3^-labelled ppGpp was prepared as described in Shyp *et al*.^47^ using H^3^-GDP as a substrate (Hartmann Analytic). The experiments were performed in three technical replicates.

## Additional Information

**How to cite this article**: Andresen, L. *et al*. Cationic bactericidal peptide 1018 does not specifically target the stringent response alarmone (p)ppGpp. *Sci. Rep.* **6**, 36549; doi: 10.1038/srep36549 (2016).

**Publisher’s note:** Springer Nature remains neutral with regard to jurisdictional claims in published maps and institutional affiliations.
